# Early Keratectomy in the Treatment of Moderate *Fusarium* Keratitis

**DOI:** 10.1371/journal.pone.0042126

**Published:** 2012-08-24

**Authors:** Hsin-Chiung Lin, Ja-Liang Lin, Dan-Tzu Lin-Tan, Hui-Kang Ma, Hung-Chi Chen

**Affiliations:** 1 Department of Ophthalmology, Chang Gung Memorial Hospital, Chang Gung University College of Medicine, Taoyuan, Taiwan; 2 Department of Internal Medicine, Chang Gung Memorial Hospital, Chang Gung University College of Medicine, Taoyuan, Taiwan; University of California, Berkeley, United States of America

## Abstract

**Purpose:**

To evaluate the treatment outcomes and costs of early keratectomy in the management of moderate *Fusarium* keratitis.

**Methodology/Principal Findings:**

Consecutive cases of culture proven *Fusarium* keratitis treated at our hospital between January 2004 to December 2010 were included in this retrospective study. There were 38 cases of moderate keratitis with infiltrates between 3 to 6 mm in diameter and depth of infiltration not exceeding the inner 1/3 of the cornea. After excluding 5 patients with incomplete follow-up data, 13 patients who received early keratectomy within 1 week of admission were compared with a group of 20 patients treated medically. The significance of the association between early keratectomy and visual acuity, progression to perforation, secondary glaucoma and cataract formation, adjuvant therapy, hospitalization days and cost were assessed. There were no differences between the keratectomy and medication groups in regards to age, sex, presence of systemic diseases, and hypopyon formation on presentation. The early keratectomy group had a shorter hospital stay than the medical therapy group. Disease duration was significantly lower in the early keratectomy group (median: 29.0 vs. 54.5 days, *P*<0.001). Median hospitalization costs per patient were lower with early keratectomy (mean ward fee: 15175.4 vs. 44159.5 NTD, *P*<0.001; mean donor fee: 0 vs. 900.0 NTD, *P*<0.001), primarily because of reductions in hospital stay. More patients in the medication group developed perforations than in the keratectomy group (20% vs. 0%, respectively) and the perforation-free rate was higher in those with early keratectomy, but the results were not statistically significant.

**Conclusions/Significance:**

Early keratectomy in moderate *Fusarium* keratitis may reduce length of hospital stay, hospital costs, and perforation rates.

## Introduction

The treatment of fungal keratitis can be challenging because the typical feathery, fluffy infiltration and endothelial plaque formation are not evident at the early stage of the disease, and the infection is often advanced on presentation due to inherent delays in arriving at an accurate diagnosis. Medical management is normally employed for the initial management of fungal keratitis, especially in mild cases where the infiltrates are located superficially, and surgical intervention is performed when medical management has failed. In advanced fungal keratitis with imminent perforation, surgical intervention in the form of therapeutic penetrating keratoplasty, conjunctival flap, or cryotherapy is often required [1]–[5].

Fungal infection has a relatively silent early phase in the cornea as compared with bacterial keratitis, as a few weeks are usually required before the infiltrate becomes evident and the stroma becomes inflamed [6], [7]. The lack of marked inflammation in the surrounding stroma permits direct visualization of the delicate, feathery branching hyphae and facilitates debulking of the fungal elements by keratectomy; there is concern regarding an increased risk of corneal perforation after early keratectomy; however, because *Fusarium* keratitis appears to be the most virulent and most common form of fungal keratitis in tropical and subtropical regions, it might be valuable to figure out the effectiveness and risk of complications of performing early keratectomy in moderate *Fusarium keratitis*.

The objectives of this study were to compare treatment outcomes, costs of care, and long-term complications in patients with moderate *Fusarium* keratitis that received early keratectomy as compared to those treated medically.

## Methods

### Patients and Treatments

The study protocol adhered to the tenets of the Declaration of Helsinki, and was approved by the Human Research Ethics Committee at Chang Gung Memorial Hospital, Taiwan (99-0576B). Based on our previous report on the treatment of *Fusarium solani* keratitis [8], corneal smears were cultured for bacteria and fungi on chocolate, 5% sheep blood, anaerobic blood agar, inhibitory mold agar (IMA), IMA supplemented with chloramphenicol and gentamicin (ICG) agar, and thioglycollate medium. External eye photographs were documented weekly, and re-culture was performed in cases of progression or poor medical response after the cessation of empiric topical antibiotics for 24 hours. At the initial encounter all patients were informed about the possibility of ulcer progression and that an operation may be needed during the disease process. In some cases, keratectomy was suggested for the purpose of obtaining a diagnostic biopsy in culture negative cases. Other patients with moderate severity disease were given the option of surgical or medical management.

Inclusion criterion was culture proven *Fusarium* infection of the cornea in patients seen between January 2004 and December 2010. Severity of keratitis was graded prospectively according to a modification of that described by Jones [1] (Table 1). The ulcer was graded as severe if the area of suppuration was >6 mm in diameter, involved the inner third of the cornea, or if perforation was imminent. Because the progression of *Fusarium* keratitis is relatively slow as compared with bacterial keratitis, we further divided non-severe keratitis into mild and moderate. Study cases were included if the corneal infiltrate was ≥3 mm but <6 mm in diameter, and the depth of involvement was not more than the inner 1/3 of the corneal thickness. Cases were excluded from analysis if <16 years of age, severe ocular surface disease was present such as Stevens-Johnson syndrome, chemical burn, or neurotrophic keratitis [9]. Subjects were followed-up for a minimum of 3 months.

**Table 1:** Clinical grading and treatment of fungal keratitis*.

Grade	Clinical Findings	Treatment
Mild	Infiltration not exceeding anterior 1/3 stroma, <3 mm in diameter	Debridement and topical antifungal medication
Moderate	Infiltration of 1/3 to 2/3 of the stroma, 3–6 mm in diameter	Debridement or superficial keratectomy, topical/with or without oral antifungal medication
Severe	Infiltration deep to inner 1/3 stroma, or >6 mm in diameter	Therapeutic keratoplasty, topical/with or without oral antifungal medication

* As described by Jones [1], with modifications.

Data collected from the medical records included age, sex, presence of systemic diseases, inciting factors, presenting symptoms, location and size of the infiltrate, presence of anterior chamber reaction, hypopyon, endothelial plaque, significant progressive pain, visual acuity (initial visual acuity was assessed at the initial patient visit, and final visual acuity was assessed upon healing of the epithelial defect), antibiotics chosen for outpatient therapy, initial medical treatment and surgical procedures after admission, length of hospital stay, disease duration, readmission, subsequent adjuvant therapy or surgery, and secondary complications.

Areas of keratitis were debrided with a No. 15 blade for microbial culture and sensitivity testing before initiation of therapy. Empiric broad-spectrum antibiotics, such as an aminoglycoside and cefazolin, ciprofloxacin, or levofloxacin were administered before culture results were obtained. Subsequent modifications in antibiotic choice and dosage depended on results of culture and sensitivity testing and clinical response. *Fusarium* keratitis was treated with topical 5% natamycin drops (Alcon Labs, Texas, USA). Superficial keratectomy was performed with a No. 57 Beaver blade, and the stromal infiltrate was removed as completely as possible. The maximum depth of keratectomy was approximately 1/2 to 2/3 of the cornea depth. In cases of severe keratitis, a corneal patch graft or amniotic membrane transplantation was used as adjuvant therapy as previously described [8], [10]. Supportive therapy included cycloplegics, analgesics, and antiglaucoma medications whenever required.

The patients were monitored until the ulcer healed, which was considered the absence of symptoms and corneal infiltrate, with decreased ciliary congestion and healed epithelial defect. Follow-up visits for a minimum of 3 months after ulcer healing were scheduled to ensure there was no recurrence of *Fusarium* keratitis.

### Cost Analysis

Component costs were estimated by reviewing the cost of treatment of a series of cases of microbial keratitis at a tertiary referral hospital. This analysis yielded values for components of treatment, i.e., hospital bed days, operating room time, and surgical team.

### Statistical Analysis

Continuous data are presented as median and inter-quartile range (IQR) except for cost data, which are presented as mean and standard deviation. The differences between groups were tested with the non-parametric Wilcoxon rank-sum test except for cost data that were tested with the independent two samples test. Categorical data are presented as the number and percentage, and the differences between groups were tested with Fisher's exact test. The primary outcome measure, perforation-free rate, was determined by the Kaplan-Meier estimates and tested with the log-rank test. As visual acuity data are not linear, they were converted to LogMAR for statistical analysis [11]. In brief, Log 20/2000 = 2 = counting fingers, and Log 20/20000 = 3 = hand motion. Cost analysis data are reported in New Taiwan Dollars (NTDs). All hypothesis tests were 2-tailed, and a value of *P*<0.05 was considered to indicate statistical significance. All statistical analyses were performed using Stat View (Abacus Concepts, Inc).

## Results

A total of 38 patients with moderate keratitis (involving 1/3 to 2/3 the stromal depth, and 3 to 6 mm in diameter) were seen during the study period. Of the 38 patients, 13 received superficial keratectomies within 1 week of admission, 20 patients received medical therapy only, and 5 patients were excluded due to follow-up of <3 months (Table 2). There were no significant differences between the two groups with respect to age, sex, involved eyes, location (central or periphery), baseline chronic diseases, smoking, and severity of the corneal ulcer. However, the baseline visual acuity of the medication group was statistically significantly worse than that of the surgery group.

**Table 2:** Baseline characteristics of patients with moderate severe *Fusarium* keratitis.

		Keratectomy group (*n* = 13)	Medication group (*n* = 20)	*P*-value
Age (year)		57.0 (48.0, 59.0)	62.0 (54.5, 67.0)	0.060
Sex	Female	8 (61.5)	7 (35.0)	0.169
	Male	5 (38.5)	13 (65.0)	
Infected Eye	OD	4 (30.8)	9 (45.0)	0.485
	OS	9 (69.2)	11 (55.0)	
Diabetes mellitus		3 (23.1)	6 (30.0)	>0.999
Hypertension		4 (30.8)	7 (35.0)	>0.999
Smoking		2 (15.4)	2 (10.0)	>0.999
Initial VA (log Mar)		1.2 (1.0, 2.0)	2.0 (1.6, 2.0)	0.033*
Area of keratitis (mm^2^)		12.5 (11.9, 16.0)	12.6 (9.5, 13.9)	0.319
Hypopyon formation		5 (38.5)	6 (30.0)	0.714

Data are presented as median (interquartile range) or number (percentage).

* Indicates a signficant difference between the two groups.

Outcomes of the two groups are shown in Table 3, and specific data of the keratectomy group are presented in Table 4. In the keratectomy group, two patients had resolution of the corneal ulcer after keratectomy without the application of natamycin. In these patients the keratitis healed with re-epithelialization before the culture grew *Fusarium* sp. Topical antibiotics were switched to natamycin after culture results were available. Five patients had progression of keratitis after keratectomy that was successfully controlled with natamycin, and no patients experienced perforation or glaucoma.

**Table 3:** Comparison of treatment methods, outcomes, and complications.

	Keratectomy group (*n* = 13)	Medication group (*n* = 20)	*P*-value
Hospital stay (d)	11.0 (9.0, 14.0)	31.5 (19.5, 49.0)	<0.001*
Disease duration (d)	29.0 (24.0, 33.0)	54.5 (37.0, 70.0)	<0.001*
Hospital cost†			
Ward fee	15175.4±7062.7	44159.5±25926.9	<0.001*
Operation room	3479.0±0	4068.3±4642.7	0.577
Donor fee	0±0	900.0±923.4	<0.001*
AMG	0 (0.0%)	4 (20.0%)	0.136
Patch graft	0 (0.0%)	4 (20.0%)	0.136
Glaucoma	0 (0.0%)	4 (20.0%)	0.136
Final VA (log Mar)	0.4 (0.2, 1.1)	2.0 (0.9, 3.0)	<0.001*
Perforation	0 (0.0%)	4 (20.0%)	0.136
Recurrence	0 (0.0%)	3 (15.0%)	0.261
Follow-up time (month)	6.0 (5.0, 12.0)	10.0 (6.0, 23.5)	0.083

Hospital stay, disease duration, final VA, and follow-up time were presented as median with IQR. Hospital costs were presented by mean ± standard deviation. Other categorical data are presented by count with percentage.

AMG, amniotic membrane graft; VA, visual acuity.

* Indicates a signficant difference between the two groups.

† New Taiwan Dollars (NTD).

**Table 4:** Data of patients treated with early keratectomy.

Case	Foreign body	Gender/age/eye/systemic disease/smoking	Keratectomy (days after admission)	Hospital stay (d)	Size of ulcer/depth	Hypopyon	Medications/culture before referral	Adjuvant therapy*	Initial VA/Final VA	Complications/follow-up time (months)
1	Vegetable	F/64/OS/H	3	9	3×4 mm	No	Gentamicin/No	No	0.1/0.4	None/6
2	UFB	F/77/OS/smoking	6	14	3.5×3 mm	Yes	Norfloxacin/No	No	CF 40 cm/0.06	None/6
3	UFB	F/62/OS/DM	3	11	4×4 mm	No	Acyclovir, Ciprofloxacin/No	No	CF 30 cm/0.08	None/7
4	NA	F/39/OS/H	2	3	4.5×3.8 mm	Yes	NA	No	CF 50 cm/0.06	None/5
5	Iron dust	M/41/OS	3	9	4×6 mm	No	Gentamicin/No	No	HM 10 cm/0.7	None/4
6	NA	M/48/OD	4	10	3.6×3.3 mm	Yes	NA	No	0.8/1.0	None/36
7	Dirt	F/37/OS	5	8	3×3 mm	No	Ciprofloxacin/No	No	0.3/0.7	None/4
8	Flower	F/56/OD	2	4	3×3 mm	No	Levofloxacin/No	No	0.4/0.5	None/3
9	Dirt	M/57/OS/H	6	23	6×4 mm	Yes	Norfloxacin/no growth	No	0.1/0.3	None/12
10	Plastic	F/57/OD/DM	6	14	3.5×3.5 mm	No	Oral famciclovir, dexan, ciprofloxacin/No	No	CF 30 cm/0.7	None/5
11	Vegetable	M/53/OS	3	20	4.5×3 mm	No	Norfloxacin/No	No	CF 1 m/0.4	None/6
12	UFB	F/59/OS	4	16	2.5×5 mm	Yes	Gentamicin/No	No	0.1/0.05	None/14
13	UFB	M/57/OD/DM, HTN/smoking	3	11	4×4 mm	No	NA/No	No	0.05/0.1	None/12

F, female; M, male; DM, diabetes mellitus; HTN, hypertension; UFB, unknown foreign body; CF, counting finger visual acuity; NA, not applicable.

* Adjuvant therapy other than early keratectomy. Disease duration: healing of epithelial defect. Final visual VA: determined upon healing of the epithelial defect.

In the medication group, eight patients exhibited stationary recovery of keratitis with medical therapy and 12 patients had progression of keratitis. Of the 12 patients with progression, four received amniotic membrane grafts as adjuvant therapy and four experienced microperforations and focal peripheral anterior synechiae and patch grafts were needed to seal the perforations. Increased intraocular pressure occurred in four patients due to inflammation of the disease process. Three patients had recurrence of keratitis that required readmission within 30 days.

### Length of stay and clinical outcomes

The median hospital stay in the keratectomy and medication groups was 11.0 vs. 31.5 days (*P*<0.001). Significantly more patients were discharged home within 10 days after superficial keratectomy; five patients in the keratectomy group had a hospital stay of <10 days, but all patients in the medication group had a hospital stay >10 days (38.5% vs. 0%, respectively, *P* = 0.005). Disease duration and hospital bed fee were significantly lower in the study group (Table 3). The costs for adjuvant surgical procedures (amniotic membrane or patch grafts) were significantly lower in the keratectomy group (mean 0 vs. 900 NTD, *P* <0.001). The ward fee was also significantly lower in the keratectomy group (mean 15175.4 vs. 44159.5 NTD, *P*<0.001). Expenses for the operating room and surgical team were comparable between the groups.

**Figure 1:**
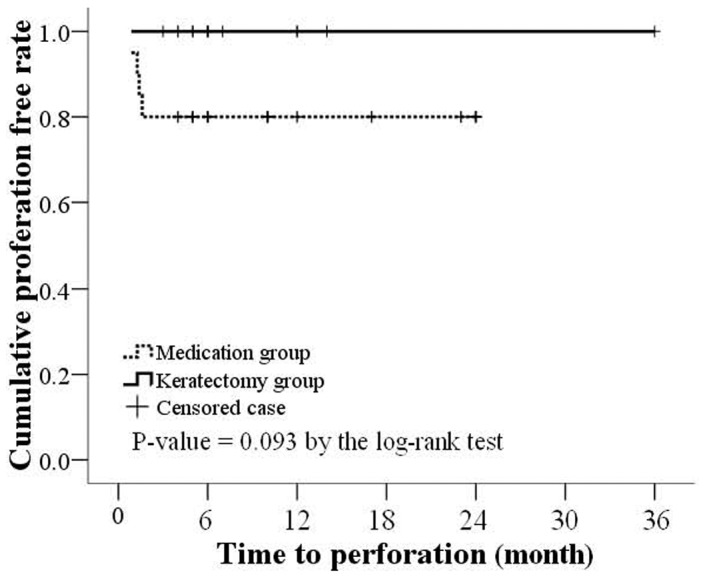
Kaplan-Meier survival analysis of corneal perforation.

Initial visual acuity ranged from hand motion to 1.0 in both groups, but final visual acuity was significantly decreased in the medication group (median log MAR 0.4 vs. 2.0, *P*<0.001). To account for the difference in baseline visual acuity, a linear regression model using the bootstrapping method with 60 repetitions was performed which showed that the final visual acuity of the keratectomy group was significantly better than the medication group (difference in logMAR = 1.05, (*P* = 0.001), a result similar to the unadjusted analysis. More patients in the medication group developed perforations than in the keratectomy group (20% vs. 0%, respectively) and the perforation-free rate was higher in those with early keratectomy, but the results did not obtain statistical significance (*P* = 0.093 by log-rank test) (Fig. 1). Four patients experienced secondary glaucoma and three had recurrence of *Fusarium* keratitis within 30 days in the medical treatment group, while no patients in the keratectomy group developed secondary glaucoma or recurrence.

## Discussion

The management of keratitis varies depending on the severity at presentation. Ophthalmologists treat suspected infectious keratitis differently with various antibiotics, debridement, and/or keratectomy, and are more likely to forgo scrapings for Gram-staining and cultures when ulcers appear less severe [1]–[4], [12]–[13]. Less severe *Fusarium* keratitis cases are treated initially with antibiotics before culture results are available, and some may resolve before the application of antifungal medications because of the resolution of the superficial lesion from scraping and the possible antifungal effects of some antibiotics [14]–[17].

**Figure 2:**
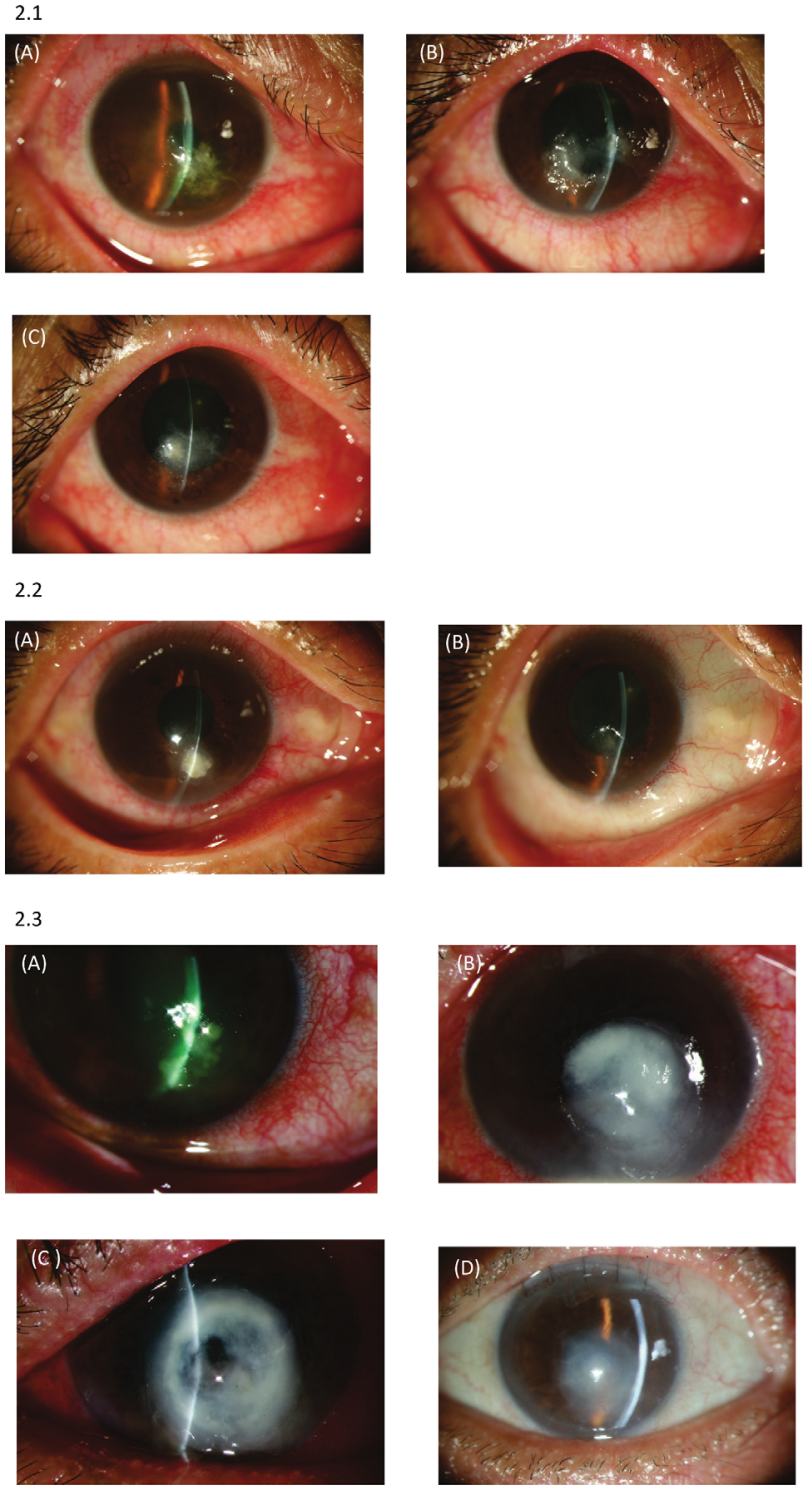
Clinical photography. 1. Clinical photographs of 57-year-old female. A) On presentation a corneal infiltrate with a feathery margin and anterior chamber inflammation were noted. Initial visual acuity was CF 30 cm. Initial debridement was performed. B) Progression of the keratitis 7 days later despite initial debridement. C) One month after superficial keratectomy and treatment with natamycin suspension. The ulcer was healed and best-corrected visual acuity was 0.7. 2. A 56-year-old female who presented with right eye pain 10 days after a foreign body injury. A) The paracentral corneal lesion appeared dry, rough, and elevated. Visual acuity 0.4. The other cornea was not inflamed. B) One week after superficial keratectomy, the lesion was healed and appeared thinned. Visual acuity 0.5. 3. A 66-year-old male. A) Central, fluffy corneal infiltration was noted on presentation. Visual acuity was hand motion. B) Three days later the infiltration had progressed with descementocele formation (C) despite topical natamycin therapy. He then received amniotic membrane transplantation as adjuvant therapy for the imminent perforation. D) Eleven months later a cataract surgery was performed and visual acuity remained counting fingers due to central scarring.

In moderate *Fusarium* keratitis, the infiltrate usually cannot be removed by scraping alone, and a superficial keratectomy with a Beaver blade or phototherapeutic keratectomy is needed in order to remove the stromal infiltrate as completely as possible [8], [18]. Debate about the treatment of moderate *Fusarium* keratitis centers around concern over the increased risk of corneal perforation after early keratectomy. Despite this concern, there are a number of reasons for performing early keratectomy in moderate *Fusarium* keratitis. There are few commercially available antifungal medications (as compared to the number available for bacterial keratitis), clinical drug sensitivity testing is not widely available, and the growth of fungi tends to be slower as compared to bacterial growth, making clinical judgment surrounding medication efficacy less efficient. The disease duration is longer in *Fusarium* keratitis, making the length of treatment and related drug toxicity a concern in the medical management of moderate *Fusarium* keratitis. In moderate *Fusarium* keratitis, the corneal infiltrates usually appear rough and dry in texture with distinct margins and little surrounding inflammation, making superficial keratectomy easy to perform. Lastly, the risk of progression of moderate to severe keratitis may occur within a few days in *Fusarium* keratitis, the most virulent species (Figure 2), leading to secondary cataract formation, glaucoma and perforation.

*Fusarium* keratitis most frequently occurs in tropical areas [19], [20]; however, an increasing prevalence has been noted in temperate climates, partly due to contact lens usage [9]. Most patients with superficial keratitis due to fungal origin respond to medical therapy, several antifungals have been found effective, and natamycin and voriconazole appear to forestall the need for surgical intervention [21], [22]. In contrast, almost 70% of patients with *Fusarium* keratitis with deep lesions do not respond to medical therapy alone. *Fusarium solani* is able to destroy an eye completely within a few weeks since the infection is usually severe; perforation, deep extension, and malignant glaucoma may supervene [8]. Rosa et al. [19] treated patients with superficial keratitis due to *Fusarium solani* with topical natamycin and those with deep lesions received topical natamycin and systemic antifungal medication. The average duration of treatment was 38 days and 22 (28%) patients ultimately required penetrating keratoplasty and enucleation was required in 1 patient. Alexandrakis et al. [23] reported that diagnostic corneal biopsy contributed significantly to the diagnosis, treatment, and outcome of patients with progressive infectious keratitis. A microorganism was isolated from 27 (82%) of 33 corneal biopsies, and *Fusarium spp* was one of the most common isolates in progressive keratitis.

The progression of *Fusarium* keratitis depends on the host as well as pathogenic factors [9], [24]–[30]. In this study, patients who received early keratectomy had shorter hospitalization stays and disease duration compared to those who did not. In moderate *Fusarium* keratitis, superficial keratectomy may aid in medical management by increasing drug penetration, by removing infected corneal tissue and subsequently reducing or eliminating the microbial load. In the present study, 13 study cases showed decreased inflammation after keratectomy at an early stage of moderate keratitis, without progression of ulcer or perforation or secondary glaucoma. Strategies for reducing perforation from fungal keratitis are important in Taiwan, where fungal keratitis accounts for 50% of corneal transplantations [31].

Surgery may be necessary when *Fusarium* keratitis responds poorly, or not at all, to medical therapy or when perforation is imminent [32], [33]. In the medical treatment group, four patients experienced microperforations and focal peripheral anterior synechiae and patch graft or therapeutic penetrating keratoplasty (PK) was needed to seal the perforation more than 2 weeks after admission, and three patients required readmission due to recurrence of *Fusarium* keratitis. Those complications of keratitis were primarily due to fungal keratitis refractory to treatment, leading to progression of a moderate *Fusarium* keratitis to a severe form. *Fusarium* keratitis is notorious for its recurrence after therapeutic PK [30], [34]. Furthermore, performance of early superficial keratectomy for moderate *Fusarium* keratitis, in lieu of corneal transplant surgery, could reduce the chance of intraocular extension during the acute infectious stage, and enable subsequent optical keratoplasty to be more successful.

Keay et al. [35] reported the major cost of treating microbial keratitis was hospital visits and hospital bed days and treating fungal keratitis is more expensive than treating bacterial keratitis. Hospital direct expenses were significantly lower in the early keratectomy group in the current study, mainly due to the decreased number of hospital days.

Possible biases and limitations of this study include that the data were examined retrospectively and that the study was conducted in a subspecialty clinic at a university hospital. Treatment was based on the personal preferences of the physicians (there were up to 8 specialty physicians treating patients during the study period) and whether or not the patient consented to surgery.

### Conclusions

In this population of Taiwanese with moderate *Fusarium* keratitis, early keratectomy was associated with decreased short-term and long-term complication rates, and required fewer financial resources to treat fungal keratitis. Early keratectomy may be useful in the treatment of patients with moderate severity *Fusarium* keratitis.

## References

[1] Jones DB (1981) Decision-making in the management of microbial keratitis. Ophthalmology 88: 814–820. 7322500 10.1016/s0161-6420(81)34943-4

[2] Forster RK, Rebell G (1975) The Diagnosis and Management of Keratomycoses II. Medical and Surgical Management. Arch Ophthalmol 93: 1134–1136. 1103806 10.1001/archopht.1975.01010020850004

[3] Meleod S, DeBacker C, Viana M (1996) Differential care of corneal ulcers in the community based on apparent severity. Ophthalmology 103: 479–484. 8600426 10.1016/s0161-6420(96)30668-4

[4] Loh AR, Hong K, Lee S, Mannis M, Acharya NR (2009) Practice Patterns in the Management of Fungal Corneal Ulcers. Cornea 28: 856–859. 19654533 10.1097/ICO.0b013e318199fa77

[5] Thomas P (2003) Current Perspectives on Ophthalmic Mycoses. Clin Microbiol Rev 16: 730–797. 14557297 10.1128/CMR.16.4.730-797.2003 PMC207127

[6] Alfonso E, Rosa RJ, Miller D (2005) Fungal keratitis. In: Krachmer JH, Mannis MJ, Holland EJ Cornea 2nd Ed Elsevier. Mosby 1 (86) 1101.

[7] Srinivasan M (2004) Fungal keratitis. Curr Opin Ophthalmol 15: 321–327. 15232472 10.1097/00055735-200408000-00008

[8] Lin HC, Chu PH, Kuo YH, Shen SC (2005) Clinical experience in managing Fusarium solani keratitis. Int J Clin Pract 59: 549–554. 15857351 10.1111/j.1368-5031.2005.00399.x

[9] Jurkunas U, Behlau I, Colby K (2009) Fungal keratitis: changing pathogens and risk factors. Cornea 28: 638–643. 19512908 10.1097/ICO.0b013e318191695b

[10] Chen H, Tan HY, Hsiao CH, Huang SCM, Lin KK, et al (2006) Amniotic membrane transplantation for persistent corneal ulcers and perforations in acute fungal keratitis. Cornea 25: 564–572. 16783145 10.1097/01.ico.0000227885.19124.6f

[11] Journal of Refractive Surgery (2009) Visual Acuity Conversion Chart/Masthead. J Cataract Refract Surg 2009 35: A4.

[12] Vajpayee RB, Dada T, Saxena R, Vajpayee M, Taylor HR, et al (2000) Study of the first contact management profile of cases of infectious keratitis: a hospital-based study. Cornea 19: 52–56. 10632009 10.1097/00003226-200001000-00011

[13] Tananuvat N, Suwanniponth M (2008) Microbial keratitis in Thailand: a survey of common practice patterns. J Med Assoc Thai 91: 316–322. 18575283

[14] Day S, Lalitha P, Haug S, Fothergill AW, Cevallos V, et al (2009) Activity of antibiotics against Fusarium and Aspergillus. Br J Ophthalmol 93: 116–119. 18952649 10.1136/bjo.2008.142364 PMC2606932

[15] Lin HC, Hsiao CH, Ma DHK, Yeh LK, Tan HY, et al (2009) Medical treatment for combined Fusarium and Acanthamoeba keratitis. Acta Ophthalmol 2009 87: 199–203. 10.1111/j.1755-3768.2008.01192.x 18507727

[16] Bhartiya P, Daniell M, Constantinou M, Islam F, Taylor H (2007) Fungal keratitis in Melbourne. Clin Experiment Ophthalmol 35: 124–130. 17362452 10.1111/j.1442-9071.2006.01405.x

[17] Munir W, Rosenfeld S, Udell I, Miller D, Karp C, et al (2007) Clinical response of contact lens-associated fungal keratitis to topical fluoroquinolone therapy. Cornea 26: 621–624. 17525664 10.1097/ICO.0b013e318033e7e1

[18] Lin CP, Chang CW, Su CY (2005) Phototherapeutic keratectomy in treating keratomycosis. Cornea 24: 262–268. 15778596 10.1097/01.ico.0000148313.78933.68

[19] Rosa RH Jr, Miller D, Alfonso EC (1994) The changing spectrum of fungal keratitis in south Florida. Ophthalmology 101: 1005–1013. 8008340 10.1016/s0161-6420(94)31225-5

[20] Wang L, Sun S, Jing Y, Han L, Zhang H, et al (2009) Spectrum of fungal keratitis in central China. Clin Experiment Ophthalmol 37: 763–771. 19878220 10.1111/j.1442-9071.2009.02155.x

[21] Tanure MA, Cohen EJ, Sudesh S, Rapuano CJ, Laibson PR (2000) Spectrum of fungal keratitis at Wills Eye Hospital, Philadelphia, Pennsylvania. Cornea 19: 307–312. 10832689 10.1097/00003226-200005000-00010

[22] Hariprasad SM, Mieler WF, Lin TK, Sponsel WE, Graybill JR (2008) Voriconazole in the treatment of fungal eye infections: a review of current literature. Br J Ophthalmol 92: 871–878. 18577634 10.1136/bjo.2007.136515

[23] Alexandrakis G, Haimovici R, Miller D, Alfonso EC (2000) Corneal biopsy in the management of progressive microbial keratitis. Am J Ophthalmol 129: 571–576. 10844046 10.1016/s0002-9394(99)00449-3

[24] Bharathi MJ, Ramakrishnan R, Meenakshi R, Shivakumar C, Raj DL (2009) Analysis of the risk factors predisposing to fungal, bacterial & Acanthamoeba keratitis in south India. Indian J Med Res 130: 749–757. 20090138

[25] Doczi I, Gyetvai T, Kredics L, Nagy E (2004) Involvement of Fusarium spp. in fungal keratitis. Clin Microbiol Infect 10: 773–776. 15355406 10.1111/j.1469-0691.2004.00909.x

[26] Furlanetto R, Andreo EG, Finotti IG, Arcieri ES, Ferreira MA, et al (2010) Epidemiology and etiologic diagnosis of infectious keratitis in Uberlandia, Brazil. Eur J Ophthalmol 10.1177/112067211002000312 20175055

[27] Galarreta DJ, Tuft SJ, Ramsay A, Dart JK (2007) Fungal keratitis in London: microbiological and clinical evaluation. Cornea 26: 1082–1086. 17893539 10.1097/ICO.0b013e318142bff3

[28] Green M, Apel A, Stapleton F (2008) Risk factors and causative organisms in microbial keratitis. Cornea 27: 22–27. 18245962 10.1097/ICO.0b013e318156caf2

[29] Vemuganti GK, Garg P, Gopinathan U, Naduvilath TJ, John RK, et al (2002) Evaluation of agent and host factors in progression of mycotic keratitis: A histologic and microbiologic study of 167 corneal buttons. Ophthalmology 109: 1538–1546. 12153808 10.1016/s0161-6420(02)01088-6

[30] Shi W, Wang T, Xie L, Li S, Gao H, et al (2010) Risk Factors, Clinical Features, and Outcomes of Recurrent Fungal Keratitis after Corneal Transplantation. Ophthalmology 117: 890–896. 20079930 10.1016/j.ophtha.2009.10.004

[31] Chen WL, Hu FR, Wang IJ (2001) Changing indications for penetrating keratoplasty in Taiwan from 1987 to 1999. Cornea 20: 141–144. 11248815 10.1097/00003226-200103000-00004

[32] Xie L, Dong X, Shi W (2001) Treatment of fungal keratitis by penetrating keratoplasty. Br J Ophthalmol 85: 1070–1074. 11520759 10.1136/bjo.85.9.1070 PMC1724109

[33] Xie L, Shi W, Liu Z, Li S (2002) Lamellar keratoplasty for the treatment of fungal keratitis. Cornea 21: 33–37. 11805504 10.1097/00003226-200201000-00008

[34] Gupta G, Feder RS, Lyon AT (2009) Fungal keratitis with intracameral extension following penetrating keratoplasty. Cornea 28: 930–932. 19654519 10.1097/ICO.0b013e31819b3213

[35] Keay L, Edwards K, Naduvilath T, Taylor H, Snibson G, et al (2006) Microbial keratitis predisposing factors and morbidity. Ophthalmology 113: 109–116. 16360210 10.1016/j.ophtha.2005.08.013

